# Complicated Meckel's Diverticulum in Children: Clinical Presentation, Diagnostic Work-Out, Surgical Approach and Postoperative Complications

**DOI:** 10.7759/cureus.12354

**Published:** 2020-12-29

**Authors:** Susan Vaabengaard, Line Andersen, Niels Qvist, Lars Rasmussen, Inge Ifaoui, Kristine Knudsen, Mark Ellebæk

**Affiliations:** 1 Surgical Department, Odense University Hospital, Odense, DNK; 2 Department of Pediatric Surgery, Rigshospitalet, Copenhagen, DNK

**Keywords:** meckel´s diverticulum, complications, surgery, diagnose

## Abstract

Introduction

Meckel’s diverticulum (MD) is the most common congenital anomaly of the gastrointestinal tract. The majority of cases are asymptomatic and in cases with complications, the diagnosis may be a challenge and the surgical approach is not obvious. The primary aim of the present study was to evaluate the diagnostic process and surgical approach in relation to clinical presentation. The secondary aim was to evaluate the severity of postoperative complications.

Methods

A two-center, retrospective analysis of all children below the age of 15 years, operated for complications to MD during the period from January 2003 to December 2016.

Results

A total of 58 patients were included. In the 40 patients presenting with an acute abdomen an average of 2.3 preoperative diagnostic investigations was performed. In only five cases an MD was recognized preoperatively. In the 18 patients presenting with rectal bleeding or melaena an average of 3.2 preoperative investigations were performed and in only one case the MD was recognized preoperatively. Laparoscopy was the surgical approach in 36 patients (62%) with a conversion in 8. Postoperative complications were seen in two patients (Clavien-Dindo II and IIIb).

Conclusion

Despite extensive diagnostic work-out an MD was recognized in only a few patients preoperatively. Laparoscopy was the surgical approach in two-thirds of the patients.

## Introduction

Meckel's diverticulum (MD) is the most common congenital anomaly of the gastrointestinal tract, with an occurrence of 2%-4% in the population [1-4]. In the majority of cases, MD usually is asymptomatic. Complications to MD may present as an acute abdomen caused by inflammation, perforation, intussusception, ileus due to adherences or as an acute rectal bleeding or melaena [4,5]. Thus, the various clinical presentation makes the diagnosis of MD a challenge [6].

The mucosal lining of MD is similar to the ileum, but it may contain ectopic colon mucosa, pancreatic tissue or gastric mucosa [7]. In cases with ectopic gastric mucosa, an ulcer may be developed, which may lead to arterial bleeding into the intestine or a perforation. Ectopic gastric mucosa may be detected by a Tc-99m-pertechnetate scintigraphy. The reported sensitivity and specificity are high in retrospective studies [8,9]. Still, to our knowledge, there are no prospective studies, which would be more reliable. Cases presenting with monosymptomatic rectal bleeding or melaena most often undergo upper and lower endoscopy as the primary diagnostic procedure and will fail the diagnosis. In cases presenting with an acute abdomen, a plain X-ray, ultrasonography or a CT-scan of the abdomen would be the preferred diagnostic method [8]. A diagnostic laparoscopy may be another option with the advantage of immediate therapy with the possibility of removal of a present MD [7].

The primary aim of the present study was to evaluate the diagnostic process and surgical approach in relation to clinical presentation. The secondary aim was to evaluate the severity of postoperative complications.

## Materials and methods

The study is a retrospective, two-center (tertiary referral centers for pediatric surgery) study of pediatric patients (≤15 years of age as defined by the Danish health authorities), who underwent surgical resection of MD between January 2003 and December 2016. Patients charts, including the diagnostic ICD-10-CM codes DQ430, DK562A, DC173, DQ430A and the pathology code WGHGD10XX, were all reviewed. A total of 58 eligible patients were identified.

Information on age, gender, clinical presentation, preoperative investigations, surgical intervention performed, postoperative course and histopathological findings were retrieved from the patient’s charts. Postoperative complications were registered according to the Clavien-Dindo classification (CDC) [10]. The patients could be divided into two groups with either acute abdomen or acute rectal bleeding or melaena according to the clinical presentation. Continuous data are given as medians and ranges.

The study was approved by the Danish Patient Safety Authority (3-3013-2196/1) and The Danish Data Protection Agency (16/43175).

## Results

There were 51 boys and 7 girls. The median age was 4.8 years (range: 6 days-14 years), and 62% of patients were aged 0-5 years. Forty patients presented with an acute abdomen without a history of rectal bleeding, and 18 presented with rectal bleeding or melaena as the only symptoms. The clinical characteristics, surgical approach and results of histological examinations are shown in Table 1.

**Table 1:** Patient characteristics, surgical approach, surgical procedure and the histological examination in children operated for a Meckel´s diverticulum (MD).

	Acute abdomen (N = 40)	Rectal bleeding (N = 18)
Boys	35	16
Girls	5	2
Median age (range)	4.3 years (6 d-14 y)	6.6 years (21 d-14 y)
Surgical approach		
Laparoscopy	23 (4 converted)	13 (4 converted)
Laparotomy	17	5
Surgical procedure		
Intestinal resection	23	5
Diverticulectomy	17	13
Histopathology (MD)		
Gastric mucosa	14	14
Gastric and pancreatic tissue	1	2
Pancreatic tissue	3	0
No ectopic tissue	20	2
No information	2	0

Acute abdomen

**Figure 1:**
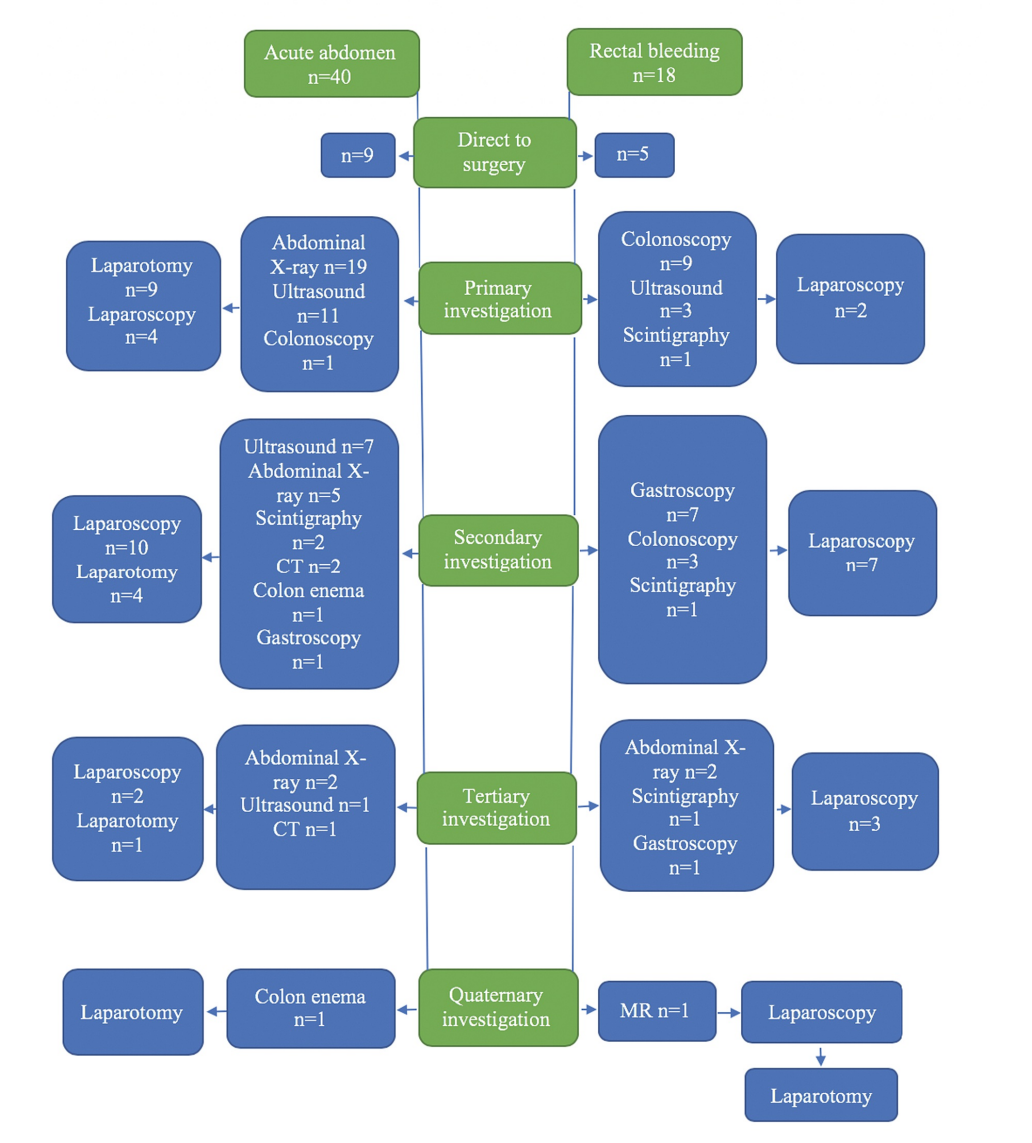
Flow-chart of preoperative investigations performed in children with complicated Meckel´s diverticulum according to clinical presentation.

Of the 40 patients, nine went directly to surgery because of the clinical presentation with peritoneal defense. The remaining 31 patients had a median of 2.3 preoperative diagnostic investigations performed (range: 1-4). In 19 patients, a plain X-ray was the primary diagnostic procedure, an abdominal ultrasonography in 11 patients and colonoscopy in one patient (Figure 1). In only five patients, an MD was recognized preoperatively, one by plain abdominal X-ray, two by a Tc-99m-pertechnetate scintigraphy, and the last two with a combination of ultrasound, plain abdominal X-ray, CT scan and a barium enema.

A total of 23 of the 40 patients underwent a primary laparoscopy (of which four were converted to laparotomy) and 17 underwent a primary laparotomy. Twenty-seven had ileus due to an invaginated MD. Thirteen patients had inflammation and in 3 cases it was complicated with perforation. Resection of the small intestine was performed in 23 and a diverticulectomy only in 17 cases. One patient was treated for postoperative paralytic ileus (CDC II). Otherwise, no other complications were recorded.

Histopathology showed ectopic gastric mucosa in 14, pancreatic tissue in three, gastric mucosa and pancreatic tissue in one. Ectopic tissue could not be demonstrated in 20 cases and in 2 cases no histopathology information was available.

Rectal bleeding or melaena (monosymptomatic)

In the 18 patients who presented with rectal bleeding or melaena a primary diagnostic laparoscopy was chosen without any preoperative investigations in five cases because am MD was suspected. The remaining 13 patients had a median of 3.2 preoperative investigations (range: 1-5). Endoscopy was the primary examination in nine cases, both upper and lower in eight cases and only lower in one case, and all with normal findings. Technetium-99m-pertechnetate scintigraphy was performed in two cases where an MD was detected in one and ultrasound in three, with normal findings in all cases. Seven patients (39%) received preoperative blood transfusion (median value for hemoglobin was 3.6 mmol/l (range: 3.1-4.7)).

The surgical approach was laparoscopy in 13 patients with conversion to laparotomy in four. Resection of the small intestine was performed in five patients and a diverticulectomy in 13 cases. In one patient with an MD located in the mesentery, the MD was not recognized until a secondary laparoscopy was performed due to ongoing rectal bleeding. The case was classified as a Clavien-Dindo IIIb complication. Otherwise, no other complications were recorded.

Histopathology showed ectopic gastric mucosa in 14 and gastric mucosa plus pancreatic tissue in two cases. Normal pathology was reported in two cases.

## Discussion

The group of patients with acute abdomen accounted for 69%. This is higher compared to other studies with a reported incidence of 42%-58% [1,8]. This could be due to patient selection as being tertial referral centers, and patients with less severe rectal bleeding may har been treated at the local hospitals.

In patients with acute abdomen, the most common diagnostic examination was an abdominal plain X-ray, which is the preferred investigation in children [11]. Ultrasound was the primary diagnostic examination in 12 patients which seems to be of limited value in the diagnosis of MD except for cases with intussusception [12]. Only two patients had a Tc-99m-pertechnetate scintigraphy and was positive in both.

In patients presenting with rectal bleeding, the majority underwent endoscopy, which may the natural primary investigation. The bleeding from an MD is often severe and may be potential life-threatening [13]. Thus, 39% of the patients in our study had preoperative blood transfusion. In this situation, the clinician may choose between a diagnostic laparoscopy or further diagnostic procedure with a Technetium 99m pertechnetate scintigraphy. The scintigraphy was positive in one of two. Despite the reported high sensitivity and specificity [9], a previous study from one of our departments revealed low values [14]. A negative scintigraphy cannot exclude a MD, and the investigation may postpone the treatment of a serious bleeding. This may advocate for a primary diagnostic laparoscopy. The drawback is, that this may result in the removal of a con-complicated MD. In our study histology revealed no pathology or ectopic tissue in two out of 18 patients. The cause of the bleeding in these two patients remained unknown but no recurrence occurred. The frequency of patients with ectopic tissue is in accordance to other studies [1,5,8,11]. 

Laparoscopy was the primary surgical approach in two-thirds of the patients with a relatively high conversion rate of 22%. Unfortunately, in one patient the MD was missed by the primary laparoscopy due to an MD located in the mesentery. Otherwise, no complications occurred. 

MD is more common in males than females, with the previously reported male to female ratio ranging from 2:1 to 4:1 [2,5,6], and most common in children younger than two years of age [7,11]. In our study, complicated MD occurred more frequently in males (88%) for which we have no clear explanation. In adults, there is also an overrepresentation of males [12]. The median age of presentation was 4.8 years (range: 6 days-14 years). In other series, median age of presentation was ranging from 3.0 to 5.3 years [1,6,8].

## Conclusions

Despite extensive diagnostic work-out, an MD was recognized in only a few patients preoperatively. Laparoscopy was the surgical approach in two-thirds of the patients but with a relatively high conversion rate. Postoperative complications were seen in two out of 58 patients 
